# On the possible advantages of combining small lineups with instructions that discourage guessing-based selection

**DOI:** 10.1038/s41598-024-64768-0

**Published:** 2024-06-19

**Authors:** Amelie Therre, Raoul Bell, Nicola Marie Menne, Carolin Mayer, Ulla Lichtenhagen, Axel Buchner

**Affiliations:** Department of Experimental Psychology, Heinrich Heine University Düsseldorf, Universitätsstraße 1, 40225 Düsseldorf, Germany

**Keywords:** Police lineups, Eyewitness identification, Two-high threshold eyewitness identification model, Lineup size, Lineup instructions, Multinomial processing tree model, Psychology, Human behaviour

## Abstract

The primary argument for including large numbers of known-to-be innocent fillers in lineups is that guessing-based selections are dispersed among a large number of lineup members, leading to low innocent-suspect identification rates. However, a recent study using the two-high threshold eyewitness identification model has demonstrated advantages of smaller lineups at the level of the processes underlying the observable responses. Participants were more likely to detect the presence of the culprit and less likely to select lineup members based on guessing in smaller than in larger lineups. Nonetheless, at the level of observable responses, the rate of innocent-suspect identifications was higher in smaller compared to larger lineups due to the decreased dispersion of guessing-based selections among the lineup members. To address this issue, we combined smaller lineups with lineup instructions insinuating that the culprit was unlikely to be in the lineup. The goal was to achieve a particularly low rate of guessing-based selections. These lineups were compared to larger lineups with neutral instructions. In two experiments, culprit-presence detection occurred with a higher probability in smaller compared to larger lineups. Furthermore, instructions insinuating that the culprit was unlikely to be in the lineup reduced guessing-based selection compared to neutral instructions. At the level of observable responses, the innocent-suspect identification rate did not differ between smaller lineups with low-culprit-probability instructions and larger lineups with neutral instructions. The rate of culprit identifications was higher in smaller lineups with low-culprit-probability instructions than in larger lineups with neutral instructions.

## Introduction

In criminal prosecution, the testimony of an eyewitness can be a valuable element to complement other evidence but also presents potential risks. False identifications by eyewitnesses have been determined to be a major reason of wrongful convictions, being involved in 63 % of DNA exoneration cases^1^. One potential problem is that the identification of a suspect is not only caused by the detection of the culprit but may also be caused by guessing-based selection. In fact, guessing-based selection occurs surprisingly frequently. For instance, in a field study with 1039 real lineups it has been found that “of all identifications made within this sample, approximately forty per cent were of fillers. These data add to a growing body of research showing that eyewitness guessing is not restricted to experimental situations with disinterested college students who know that their choices carry no consequences”[p. 264^2^]. A possible measure to reduce the rate with which an innocent suspect is identified from a lineup based on guessing is to include a large number of known-to-be-innocent fillers who share relevant characteristics with the suspect.

Provided that the lineup is fair, that is, provided that the suspect does not stand out from the fillers such that it is impossible to distinguish the suspect from the fillers without relying on memory for the culprit’s appearance^3^, a larger lineup has the advantage over a smaller lineup that guessing-based selection, if it occurs, is dispersed among a larger number of lineup members (including known-to-be-innocent fillers, therefore often described as “filler siphoning”^4,5^). Consequently, a larger lineup reduces the probability that guessing-based selection leads to the identification of the suspect among the fillers compared to a smaller lineup. Legal requirements for the number of fillers in lineups vary across jurisdictions. In addition to the suspect, lineups typically comprise five fillers in the United States^6^, at least seven in Germany^7^, eight in Great Britain^8^ and nine in Canada^9^ (for an overview, see^10^). Consequently, the probability of sampling the suspect among the lineup members in case of a guessing-based selection is 1 ÷ 6 in the United States, at most 1 ÷ 8 in Germany, 1 ÷ 9 in Great Britain and 1 ÷ 10 in Canada. If a lineup member is selected based on guessing, the smaller sampling probability associated with a larger number of fillers can be said to protect a suspect from being falsely identified^5,11,12^. This may be counted as an advantage of larger lineups over smaller lineups. However, it has been pointed out that larger lineups compared to smaller lineups are associated not only with a lower rate of innocent-suspect identifications, but also with a lower rate of culprit identifications—a cost–benefit-tradeoff^13^. Some researchers even found that larger lineups lead to a lower discriminability between culprits and innocent suspects compared to smaller lineups^14^ while others found no effect of lineup size on discriminability^15,16^ or raw identification rates^17^.

**Figure 1:**
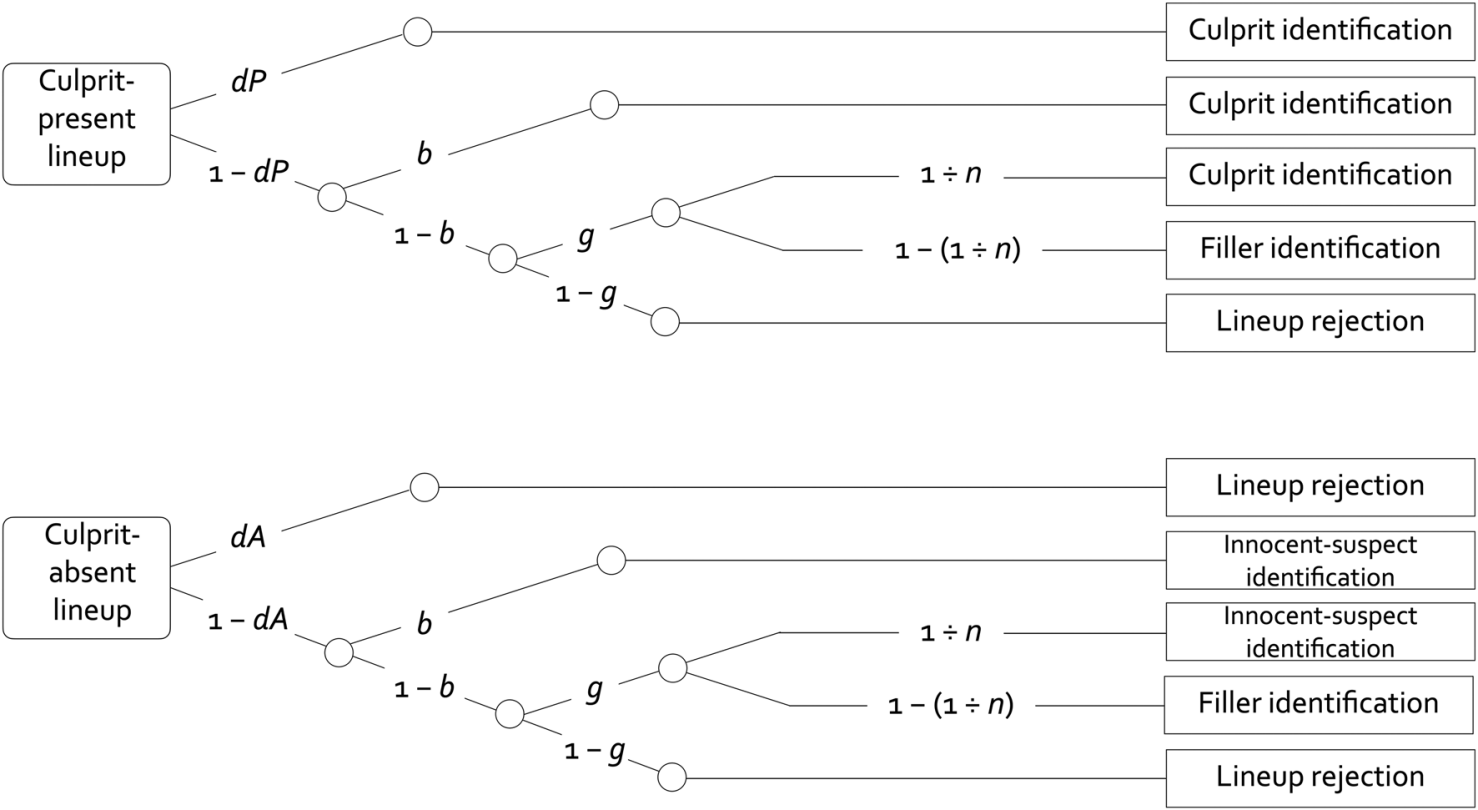
Rounded rectangles on the left represent the two types of possible lineups: culprit-present lineups and culprit-absent lineups. Rectangles to the right represent the observable response categories. Letters along the branches stand for the parameters that represent the latent processes (*dP*: probability of culprit-presence detection; *dA*: probability of culprit-absence detection; *b*: probability of biased suspect selection; *g*: probability of guessing-based selection). The constant *n* represents the number of persons in the lineup and 1 ÷ *n* represents the probability of sampling the culprit (upper tree) or the innocent suspect (lower tree) if guessing-based selection occurs.

These findings and considerations pertain to overall evaluations of eyewitness’s observable responses, that is, the rates of innocent-suspect and culprit identifications. However, the effects of lineup size on innocent-suspect and culprit-identification rates may result from various latent detection-based and non-detection-based processes. Here the two-high threshold (2-HT) eyewitness identification model^3,18–21^ is used to separately measure the latent processes underlying observable responses to lineups. The 2-HT eyewitness identification model (Fig. 1) has been successfully validated both in a series of dedicated validation experiments^20^ and by reanalyzing published data obtained in various laboratories^3^. The 2-HT eyewitness identification model belongs to the class of multinomial processing tree models. Multinomial processing tree models are easy-to-use measurement models that have been applied to many domains in cognitive research^22–25^. Multinomial processing tree models imply that overt responses result from latent processes that occur with certain probabilities^25^. The probabilities with which these processes occur are represented by model parameters that can be compared statistically by means of easy-to-use software such as *multiTree*^26^.

The 2-HT eyewitness identification model comprises all response categories that may occur when eyewitnesses try to identify a culprit from a lineup. In culprit-present lineups (see the rounded rectangle on the left side of the upper tree in Fig. 1), these response categories are culprit identifications, filler identifications and lineup rejections (see the rectangles on the right side of the upper tree in Fig. 1). In culprit-absent lineups (see the rounded rectangle on the left side of the lower tree in Fig. 1), these response categories are innocent-suspect identifications, filler identifications and lineup rejections (see the rectangles on the right side of the lower tree in Fig. 1).

In a culprit-present lineup (upper tree in Fig. 1), culprit-presence detection occurs with probability *dP*, as a result of which the culprit is identified. If the presence of the culprit is not detected, which occurs with probability 1 − *dP*, the culprit can still be selected as a consequence of non-detection-based processes. Biased selection of the culprit may occur with probability *b* if the lineup is unfair, for example, if the culprit stands out from the other members of the lineup ﻿based on physical appearance or other distinct characteristics of the suspect’s photo. With the conditional probability 1 − *b*, no biased suspect selection occurs. In this case, the eyewitness may still select one of the lineup members based on guessing with the conditional probability *g*. The probability with which this process leads to the selection of the culprit is given by the sampling probability 1 ÷ *n*, where *n* is a constant representing the lineup size. For instance, in a lineup with *n* = 6 persons, guessing-based selection will lead to the selection of the culprit with a probability of 1 ÷ 6 ≅ 0.17. With the complementary probability of 1 − (1 ÷ *n*) = 5 ÷ 6 ≅ 0.83, guessing-based selection will lead to the selection of a filler. A culprit-present lineup is falsely rejected if neither culprit-presence detection (1 − *dP*) nor biased selection (1 − *b*) nor guessing-based selection (1 − *g*) occur.

In a culprit-absent lineup (lower tree in Fig. 1), parameter *dA* reflects the probability of detecting that the culprit is not in the lineup, resulting in a correct rejection of the lineup. With probability 1 − *dA*, culprit-absence detection does not occur. In this case, biased suspect selection occurs with the conditional probability *b*, resulting in a selection of the innocent suspect. In case of no biased suspect selection (1 − *b*), guessing-based selection occurs with the conditional probability *g*, in which case the probability of selecting the innocent suspect is given by the sampling probability 1 ÷ *n* while the probability of selecting a filler is given by 1 − (1 ÷ *n*). The culprit-absent lineup is correctly rejected if neither culprit-absence detection (1 − *dA*) nor biased selection (1 − *b*) nor guessing-based selection (1 − *g*) occur.

When the 2-HT eyewitness identification model was used to evaluate the effects of lineup size on the latent processes underlying eyewitnesses’ responses to lineups^19^, interesting findings emerged on which we build here. Reducing the size of lineups (from six to three or from five to two persons) resulted in two remarkable outcomes: The probability of culprit-presence detection (represented by model parameter *dP*) was significantly higher and the probability of guessing-based selection (represented by model parameter *g*) was significantly lower in smaller compared to larger lineups. The model-based analysis thus revealed a favorable aspect of smaller compared to larger lineups at the level of the latent processes in that it seems preferable if observable eyewitness responses to lineups result from a detection process rather than from guessing-based processes.

However, an unfavorable outcome associated with smaller compared to larger lineups was obtained at the level of observable responses: The rate of innocent-suspect identifications was higher in smaller compared to larger lineups^19^. To see why this occurred, it is useful to look at the lower tree in Fig. 1. Here the model structure exposes the interplay of the latent processes behind the changes in observable responses in culprit-absent lineups when the lineup size *n* is reduced. For simplicity, let us assume that eyewitnesses do not detect the absence of the culprit (*dA* = 0) and that the lineup is perfectly fair (*b* = 0). In this case, the probability of the innocent suspect being identified as the culprit would be given by *g* × (1 ÷ *n*). Seen in isolation, a reduced probability of guessing-based selection (parameter *g*) in smaller compared to larger lineups should lead to a reduced rate with which innocent suspects are identified. However, if guessing-based selection occurs, the sampling probability that determines whether innocent suspects are identified is given by the term 1 ÷ *n*, where *n* represents the lineup size. A decrease in lineup size *n* implies that the term 1 ÷ *n* increases. For instance, 1 ÷ *n* is doubled from 0.17 in six-person lineups to 0.33 in three-person lineups. The smaller probability of guessing-based selection (parameter *g*) in smaller compared to larger lineups did not compensate for this considerable increase in the probability of randomly sampling the innocent suspect (1 ÷ *n*), leading to a net increase in the innocent-suspect identification rate in smaller compared to larger lineups*.* This is why, despite a reduction in parameter *g*, there was still a higher rate of innocent-suspect identifications in smaller compared to larger lineups^19^.

While the higher rate of innocent-suspect identifications is a clear disadvantage of smaller compared to larger lineups, the structure of the model illustrated in Fig. 1 helps finding a potential remedy: At the level of the latent processes, the innocent-suspect identification rate is strongly affected by the probability of guessing-based selection reflected in parameter *g*. Therefore, the disadvantage of smaller compared to larger lineups at the level of the innocent-suspect identification rates should become lower if people are discouraged from making guessing-based selections. It seems even possible that the rate of innocent-suspect identifications in smaller lineups reaches the rate of innocent-suspect identifications in larger lineups or perhaps even a lower rate. For this to occur it would be necessary to reduce an eyewitness’s tendency to make a guessing-based selection even below the level achieved by reducing the lineup size alone. Here it is useful that the probability of guessing-based selection can be manipulated without affecting the other processes specified in the 2-HT eyewitness identification model simply by applying appropriate lineup instructions^20^. Specifically, instructions insinuating that the culprit is unlikely to be in the lineup have been found to reduce parameter *g* without affecting the other model parameters^20^. If instructions insinuating that the culprit is unlikely to be in the lineup effectively reduce the probability of guessing-based selection in smaller lineups, then smaller lineups combined with such instructions might have no strong disadvantage in the innocent-suspect-identification rates compared to larger lineups without such instructions. This prediction was tested in the present experiments.

Apart from the unfavorable outcome of a higher rate of innocent-suspect identifications in smaller compared to larger lineups, there was also an unambiguously favorable outcome at the level of observable responses: The rate of culprit identifications was higher in smaller compared to larger lineups^19^. This is to be expected because the dominant determinant of a higher culprit identification rate in smaller compared to larger lineups is the higher probability of culprit-presence detection (parameter *dP*) in smaller compared to in larger lineups^19^. This is why we expected the rate of culprit identifications to be higher in smaller compared to larger lineups in the present experiments.

Parallel to the previous study^19^ which we build on here, three-person lineups were compared to six-person lineups. The lineup size used in the latter condition corresponds to the typical lineup size in the United States^6^. Within each lineup-size condition, about half of the participants received instructions insinuating that the culprit was unlikely to be in the lineup (henceforth low-culprit-probability instructions), which are known to reduce guessing-based selection without affecting culprit-presence detection^20^. The other half of the participants received neutral instructions emphasizing that the culprit may or may not be in the lineup. Such neutral instructions are officially recommended for police lineups^12^. We consider the combination of six-person lineups with neutral instructions to be the standard of comparison. The combination of three-person lineups with low-culprit-probability instructions was compared to this standard in terms of innocent-suspect identification rates and culprit identification rates.

Before testing the novel predictions pertaining to the level of observable responses, it is important first to test whether the previously found effects of lineup size^19^ and of low-culprit-probability instructions^20^ are robust and can be replicated. In case of a successful replication the probability of culprit-presence detection (parameter *dP*) should be higher and the probability of guessing-based selection (parameter *g*) should be lower in three-person lineups than in six-person lineups. Also, low-culprit-probability instructions should lead to a lower probability of guessing-based selection (parameter *g*) than neutral instructions.

As mentioned above, the two novel predictions that were tested in the present study pertain to the level of observable responses. Both predictions were derived from the analysis of the underlying detection-based and non-detection-based processes as measured by the 2-HT eyewitness identification model. First, the hypothesis was tested that three-person lineups with low-culprit-probability instructions should be associated with a low rate of innocent-suspect identifications, ideally at least as low as the rate observed in six-person lineups with neutral instructions (the standard of comparison). Second, the hypothesis was tested that three-person lineups with low-culprit-probability instructions should be associated with a higher rate of culprit identifications compared to the standard of comparison, that is, the six-person lineups with neutral instructions. To test these hypotheses, we conducted two experiments. Given that both sequential and simultaneous lineups are used in jurisdictions around the world^10^, we used both types of lineup formats. Specifically, sequential lineups were used in Experiment 1 and simultaneous lineups were used in Experiment 2, which served as a conceptual replication of Experiment 1.

## Experiment 1

### Method

#### Participants

Participants were recruited using the Horizoom research panel (www.horizoom-panel.de). Of the 1063 datasets of participants who had given their informed consent, 25 had to be excluded because participants had not passed the attention check (see below), 3 had to be excluded because of duplicate participation and 29 had to be excluded because participants had not completed the experiment or withdrew their consent. Consequently, datasets of 1006 participants were included in the analysis. Of these participants 528 identified as male, 475 as female and 3 as non-binary. Participants’ ages ranged from 18 to 85 years (*M* = 51). Participants were randomly assigned to one of the four experimental groups. A total of 252 participants responded to three-person lineups with low-culprit-probability instructions, 255 participants responded to three-person lineups with neutral instructions, 255 participants responded to six-person lineups with low-culprit-probability instructions and 244 participants responded to six-person lineups with neutral instructions. A sensitivity analysis with G*Power^27^ showed that given *N* = 1006 participants and four responses per participant, error probabilities of α = β = 0.05 and *df* = 1 for tests of parameter equality across two groups, effects as small as *w* = 0.06 could be detected.

#### Ethics statement

The ethics committee of the Faculty of Mathematics and Natural Sciences at Heinrich Heine University Düsseldorf has granted ethical approval for the experiments reported here. The experiments were conducted in compliance with the Declaration of Helsinki. A requirement for taking part was the participant’s declaration of informed consent prior to the experiment. Before the staged-crime video, participants were informed that they would see a video including physical and verbal violence. Participants were asked to continue the study only if they agreed to watch such a video.

#### Materials and procedure

Materials and procedure were the same as those used in earlier experiments^3,18–21^. The experiment was conducted online and was implemented in *SoSci Survey*^28^ (www.socisurvey.de). Participation was possible with a desktop or laptop computer. Participants had to be 18 years old or older (a legal requirement in Germany).

After having given their informed consent, participants provided sociodemographic data. Subsequently, participants saw one of two staged-crime videos (henceforth Video 1 and Video 2). While the actors differed between videos, the events shown as well as their sequence and timing were the same in both videos. In essence, four men dressed in fan clothing of the German soccer club FC Bayern München were the culprits who physically and verbally abused a man dressed in fan clothing of a rival soccer club, Borussia Dortmund, at a bus stop. Actors portraying the same character were selected to be similar in ﻿body shape, hair color and hairstyle, that is, the actor portraying Character A in Video 1 was similar to the actor portraying Character A in Video 2, the actor portraying Character B in Video 1 was similar to the actor portraying Character B in Video 2 and so on. The videos were presented at a resolution of 885 × 500 pixels and lasted about 130 s.

The video was followed by an attention-check question requiring participants to indicate the role of the protagonists in the video (with soccer fans being the correct option amidst nine distractor options such as knights, musicians and politicians). Next, participants were informed that they had to identify the FC Bayern München hooligans in a series of photo lineups. All participants received the following instructions (the original was in German):

“In the film, you saw Bayern München hooligans. Now we want you to identify them. To do this, we will show you several lineups. In each lineup, you will see a series of faces. You will be asked to indicate whether one of the people in the lineup is one of the Bayern München hooligans.”

Participants in the groups with low-culprit-probability instructions also received the following instructions:

“It is **unlikely** that one of the culprits is in the lineup. Therefore, you should select the ‘Yes, was present’ button that belongs to the recognized face only if you are very certain that you have recognized the right person. Otherwise, click on the ‘No, this person was not present’ button.”

In contrast, participants in the groups with neutral instructions received the following instructions:

“It is also possible that no one in the lineup is one of the Bayern München hooligans. If you recognize someone, click on the ‘Yes, was present’ button that belongs to the recognized face. Otherwise, click on the ‘No, this person was not present’ button.”

Next, four sequential lineups were shown. Depending on the lineup size, the lineups included the facial photos of one suspect and either two or five fillers. The photos were presented one at a time. For each photo, participants had to decide whether or not it depicted one of the culprits by clicking either on a button labeled “Yes, was present” below the person’s photo or on a button labeled “No, this person was not present”. It was possible to choose more than one person in each lineup. As in prior studies^3,18–21^, the last selection was considered to be a revision of any earlier selections and was used in the analyses.

In two lineups, a randomly selected culprit was present. In the other two lineups, an innocent suspect was present. ﻿The photos of the innocent suspects were photos of the actors from the video participants had not seen. For instance, if participants had seen Video 1 and the two randomly selected culprits from Video 1 were culprits portraying Characters B and C, then the culprits portraying Characters A and D from Video 2 were selected as innocent suspects in the culprit-absent lineups. This crossed-lineup procedure is identical to the one applied in prior studies^18–21^ and ensures that the photos of culprits and innocent suspects (taken right after the videos had been shot) differ to the same degree from the photos of the fillers (taken from a face database^29^ with the goal to resemble one of the culprits in body shape, hair color and hairstyle). This is parallel to the real world where the photos of the suspects (whose status of being innocent or guilty is unknown to the police) are often taken from a different source (e.g., from social media) than the photos of the fillers which are typically obtained from face databases. The order of the lineups and the positions of all photos in a lineup, including those of the culprit or innocent suspect, were randomly determined. For three-person lineups, random two-filler subsets were created from the set of five fillers used for the six-person lineups.

Participants in the groups with low-culprit-probability instructions were provided with the following reminder prior to each lineup: “It is unlikely that one of the culprits is in the lineup. Please choose someone only if you are very certain.” Participants in the groups with neutral instructions did not receive a reminder.

After having responded to all lineups, participants were asked to reaffirm their consent to the use of their data, debriefed, thanked for their participation and redirected to the panel provider to receive their monetary compensation.

### Results

The response frequencies obtained in Experiment 1 are presented in Table 1 (together with those of Experiment 2). The files with the raw frequency data and the equation files needed for the model-based analyses are available at https://osf.io/gcm8x/.

**Table 1:** Response frequencies in culprit-present and culprit-absent lineups for each combination of the lineup-size variable (three-person lineups vs. six-person lineups) and the instruction variable (low-culprit-probability instructions vs. neutral instructions) in Experiments 1 and 2.

Lineup size	Instructions	Culprit-present lineups			Culprit-absent lineups		
		Culprit identifications	Filler identifications	Lineup rejections	Innocent-suspect identifications	Filler identifications	Lineup rejections
Experiment 1 – sequential lineups							
Three	Low-culprit-probability	212	92	200	73	124	307
	Neutral	260	122	128	131	166	213
Six	Low-culprit-probability	126	166	218	52	205	253
	Neutral	152	214	122	72	252	164
Experiment 2 – simultaneous lineups							
Three	Low-culprit-probability	217	62	227	78	91	337
	Neutral	255	93	170	94	140	284
Six	Low-culprit-probability	149	99	270	39	116	363
	Neutral	186	130	196	62	177	273

#### Latent processes: Effects on parameters *dP* and *g* of the 2-HT eyewitness identification model

All model-based analyses were conducted using *multiTree*^26^. Four instances of the model illustrated in Fig. 1 were needed to analyze the data, one instance for each combination of the lineup-size variable (three-person lineups vs. six-person lineups) and the instruction variable (low-culprit-probability instructions vs. neutral instructions). The term (1 ÷ *n*) which represents the probability of randomly sampling the suspect in case of guessing was set to 0.33333 for data obtained with three-person lineups (approximating 1 ÷ 3) and 0.16667 for data obtained with six-person lineups (approximating 1 ÷ 6).

To arrive at a testable base model, restrictions were applied to the 2-HT eyewitness identification model that were identical to those used in previous studies in which lineup size was manipulated^19^. Specifically, parameter *b* was set to be equal across all four instances of the model given that the lineups were composed of the same suspects and fillers in all groups such that there was no obvious reason as to why lineup fairness should differ between groups. Parameter *dA* was also set to be equal across all instances of the model because the manipulations used here were clearly different from those known to affect the probability of culprit-absence detection^3,20^. The base model with these restrictions fit the data, *G*^2^(6) = 8.04, *p* = 0.235, providing support for the assumptions implemented in the base model. Parameters *b* and *dA* were estimated to be 0.04 (*SE* = 0.01) and 0.03 (*SE* = 0.03), respectively.

The estimates of parameters *dP* and *g* are presented in Table 2. One goal of the present research was to test whether the previously found effects of lineup size on culprit-presence detection and guessing-based selection can be replicated^19^. Table 2 shows that the probability of culprit-presence detection (parameter *dP*) was higher in three-person lineups than in six-person lineups. To test whether this difference was statistically significant, we imposed on the base model the additional restriction that parameter *dP* did not differ between three-person and six-person lineups, separately for the low-culprit-probability-instruction group and the neutral-instruction group. The decrease in model fit caused by this additional restriction compared to the fit of the base model was statistically significant for both the low-culprit-probability-instruction group, Δ*G*^2^(1) = 21.04, *p* < 0.001, and the neutral-instruction group, Δ*G*^2^(1) = 20.12, *p* < 0.001, implying that the equality restriction is incompatible with the data. This leads to the conclusion that the probability of culprit-presence detection is indeed higher in three-person lineups than in six-person lineups. Table 2 also shows that the probability of guessing-based selection (parameter *g*) was lower in three-person lineups than in six-person lineups. To test whether this difference was statistically significant, we imposed on the base model the additional restriction that parameter *g* did not differ between three-person and six-person lineups, separately for the low-culprit-probability-instruction group and the neutral-instruction group. The decrease in model fit caused by this additional restriction compared to the fit of the base model was statistically significant for both the low-culprit-probability-instruction group, Δ*G*^2^(1) = 15.42, *p* < 0.001, and the neutral-instruction group, Δ*G*^2^(1) = 12.81, *p* < 0.001, implying that the equality restriction is incompatible with the data. This leads to the conclusion that the probability of guessing-based selection is indeed lower in three-person lineups than in six-person lineups. It can thus be concluded that the previously found effects of smaller compared to larger lineups on the processes underlying eyewitnesses’ responses to lineups^19^ are robust and can be replicated.

**Table 2:** Parameter estimates of parameter *dP* (representing the probability of culprit-presence detection) and of parameter *g* (representing the probability of guessing-based selection) in Experiment 1.

Lineup Size	Instructions	Estimates of parameter *dP*		Estimates of parameter *g*	
Three	Low-culprit-probability	0.31	(0.03)	0.39	(0.02)
	Neutral	0.37	(0.03)	0.59	(0.02)
Six	Low-culprit-probability	0.15	(0.02)	0.49	(0.02)
	Neutral	0.19	(0.03)	0.68	(0.02)

Values in parentheses are standard errors.

Next, we tested whether low-culprit-probability instructions reduce the probability of guessing-based selection (parameter *g*) compared to neutral instructions. Table 2 shows that the estimate of parameter *g* is lower in the low-culprit-probability-instruction group than in the neutral-instruction group. To test whether this difference is statistically significant, we imposed on the base model the additional restriction that parameter *g* did not differ as a function of whether low-culprit-probability or neutral instructions were used, separately for the three-person-lineup group and the six-person-lineup group. The decrease in model fit caused by this additional restriction compared to the fit of the base model was statistically significant for both the three-person-lineup group, Δ*G*^2^(1) = 52.24, *p* < 0.001, and the six-person-lineup group, Δ*G*^2^(1) = 55.75, *p* < 0.001, implying that the equality restriction is incompatible with the data. This leads to the conclusion that low-culprit-probability instructions lead to a lower probability of guessing-based selection compared to neutral instructions. As an aside, manipulations aimed at changing the probability with which guessing-based selection occurs should not affect other model parameters such as parameter *dP*^3,20^. The restriction that parameter *dP* did not differ as a function of whether low-culprit-probability or neutral instructions were used did not lead to a statistically significant decrease in model fit compared to the fit of the base model for both the three-person-lineup group, Δ*G*^2^(1) = 2.27, *p* = 0.132, and the six-person-lineup group, Δ*G*^2^(1) = 1.95, *p* = 0.162, implying that the equality restriction is compatible with the data. This leads to the conclusion that culprit-presence detection does not differ as a function of the lineup instructions. It can thus be concluded that the previously found effects of low-probability instructions on the latent processes underlying eyewitnesses’ responses to lineups^20^ are robust and can be replicated.

#### Observable responses: Effects on the rates of innocent-suspect identifications and culprit identifications

**Figure 2:**
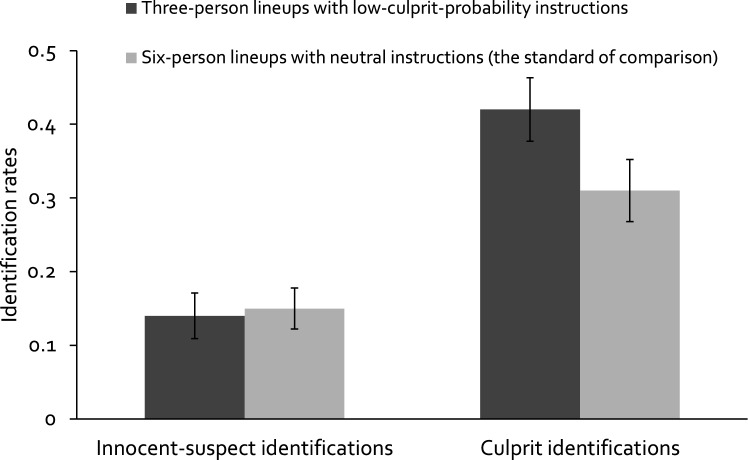
Rates of innocent-suspect identifications (left side) and rates of culprit identifications (right side) for three-person lineups with low-culprit-probability instructions and for six-person lineups with neutral instructions (the standard of comparison) in Experiment 1. Error bars represent the 95 % confidence intervals.

Given these successful replications we next tested whether combining three-person lineups with low-culprit-probability instructions would lead to a low rate of innocent-suspect identifications, ideally at least as low as the rate observed in six-person lineups with neutral instructions (the standard of comparison). The rates of innocent-suspect identifications in three-person lineups with low-culprit-probability instructions and six-person lineups with neutral instructions are presented in Fig. 2 (left side). Three-person lineups with low-culprit-probability instructions were associated with a rate of innocent-suspect identifications close to the rate observed in six-person lineups with neutral instructions. A two-proportion *z*-test showed that there was no statistically significant difference between these groups in the rate of innocent-suspect identifications in Experiment 1, *z* = 0.12, *p* = 0.904. This leads to the conclusion that the two rates are equal.

Finally, we tested whether three-person lineups combined with low-culprit-probability instructions would lead to higher rates of culprit identifications compared to the rates observed in six-person lineups with neutral instructions. The rates of culprit identifications are also presented in Fig. 2 (right side). Three-person lineups with low-culprit-probability instructions were associated with a higher rate of culprit identifications than six-person lineups with neutral instructions. A two-proportion *z*-test showed that the difference between these groups in the rate of culprit identifications was statistically significant, *z* = 3.57, *p* < 0.001. This leads to the conclusion that the rate of culprit identifications is higher for three-person lineups with low-culprit-probability instructions compared to six-person lineups with neutral instructions.

### Discussion

The probability of culprit-presence detection was significantly higher and the probability of guessing-based selection was significantly lower in smaller compared to larger lineups. This replicates earlier findings^19^. In addition, low-culprit-probability instructions led to a significantly smaller probability of guessing-based selection than neutral instructions. In contrast, the type of instructions did not affect the probability of culprit-presence detection. This, too, replicates earlier findings^3,20^. It can be concluded that instructions implying that the culprit is unlikely to be in the lineup effectively discourage guessing-based selection.

Two novel predictions were derived about how small lineups combined with low-culprit-probability instructions should affect observable responses compared to six-person lineups with neutral instructions (the standard of comparison). First, combining three-person lineups with low-culprit-probability instructions should be associated with a low rate of innocent-suspect identifications, ideally at least as low as the rate observed in six-person lineups with neutral instructions. Second, three-person lineups with low-culprit-probability instructions should be associated with a higher rate of culprit identifications compared to six-person lineups with neutral instructions. The fact that both of these predictions were confirmed demonstrates that a deeper understanding of the latent processes underlying eyewitness responses can successfully lead to useful predictions about observable responses.

However, before drawing any firm conclusions it seemed important to test the robustness of these findings in a conceptual replication study, which was the purpose of Experiment 2. Given that not only sequential, but also simultaneous lineups are used in jurisdictions around the world^10^, Experiment 2 was parallel to Experiment 1 except that simultaneous lineups were used. We expected to replicate the findings of Experiment 1 in Experiment 2.

## Experiment 2

### Method

#### Participants

Participants were recruited using the Horizoom research panel (www.horizoom-panel.de). Of the 1105 datasets of participants who had given their informed consent, 21 had to be excluded because participants had not passed the attention check, 1 had to be excluded because of duplicate participation and 56 had to be excluded because participants had not completed the experiment or withdrew their consent. Consequently, datasets of 1027 participants, none of whom had participated in Experiment 1, were included in the analyses. Of these participants, 564 identified as male, 458 as female and 5 as non-binary. Participants’ ages ranged from 18 to 84 years (*M* = 49). Participants were randomly assigned to one of the four experimental groups. A total of 253 participants responded to three-person lineups with low-culprit-probability instructions, 259 participants responded to three-person lineups with neutral instructions, 259 participants responded to six-person lineups with low-culprit-probability instructions and 256 participants responded to six-person lineups with neutral instructions. A sensitivity analysis with G*Power^27^ showed that given *N* = 1027 participants and four responses per participant, error probabilities of α = β = 0.05 and *df* = 1 for tests of parameter equality across two groups, effects as small as *w* = 0.06 could be detected.

#### Materials and procedure

Materials and procedure were identical to those of Experiment 1 except that simultaneous rather than sequential lineups were used. For each lineup, all six photos were shown next to each other at the same time. Participants could either select one person by clicking on a button labeled “Yes, was present” below the person’s photo or reject the lineup by clicking on a button labeled “No, none of these persons was present”.

### Results

The response frequencies obtained in Experiment 2 are presented in Table 1 (together with those of Experiment 1). The files with the raw frequency data and the equation files needed for the model-based analyses are available at https://osf.io/gcm8x/.

#### Latent processes: Effects on parameters *dP* and *g* of the 2-HT eyewitness identification model

The term (1 ÷ *n*) was set to 0.33333 and 0.16667 for data obtained with three-person lineups and six-person lineups, respectively. The same restrictions as in Experiment 1 were used to arrive at a base model which fit the data, *G*^2^(6) = 3.40, *p* = 0.757. Parameters *b* and *dA* were estimated to be 0.05 (*SE* = 0.01) and 0.04 (*SE* = 0.05), respectively.

The estimates of parameters *dP* and *g* are presented in Table 3. The probability of culprit-presence detection (parameter *dP*) was higher in three-person lineups than in six-person lineups. Imposing on the base model the additional restriction that parameter *dP* did not differ between three-person and six-person lineups led to a significant decrease in model fit for both the low-culprit-probability-instruction group, Δ*G*^2^(1) = 11.97, *p* < 0.001, and the neutral-instruction group, Δ*G*^2^(1) = 6.89, *p* = 0.009, implying that the equality restriction is incompatible with the data. This leads to the conclusion that the probability of culprit-presence detection is higher in three-person lineups than in six-person lineups. The probability of guessing-based selection (parameter *g*) was not lower in three-person lineups than in six-person lineups. Imposing on the base model the additional restriction that parameter *g* did not differ between the three-person and six-person lineups did not lead to a significant decrease in model fit for both the low-culprit-probability-instruction group, Δ*G*^2^(1) = 0.22, *p* = 0.637, and the neutral-instruction group, Δ*G*^2^(1) = 0.06, *p* = 0.815, implying that the equality restriction is compatible with the data. In sum, then, the effects of smaller compared to larger lineups on the latent processes underlying eyewitnesses’ responses to lineups reported in previous research^19^ were replicated with respect to culprit-presence detection but not with respect to guessing-based selection. However, in those earlier results the difference in the estimates of parameter *g* between three-person lineups and six-person lineups was descriptively smaller for simultaneous lineups than for sequential lineups. From this pattern of findings, it seems possible to infer that the effect of lineup size on the probability of guessing-based selection may be relatively small and is therefore not reliably observed in simultaneous lineups. However, this is of course only a post-hoc speculation and the reasons as to why the effect of lineup size on the probability of guessing-based selection might be comparatively small in simultaneous lineups are currently unknown.

**Table 3:** Parameter estimates of parameter *dP* (representing the probability of culprit-presence detection) and of parameter *g* (representing the probability of guessing-based selection) in Experiment 2.

Lineup Size	Instructions	Estimates of parameter *dP*		Estimates of parameter *g*	
Three	Low-culprit-probability	0.33	(0.03)	0.30	(0.02)
	Neutral	0.37	(0.03)	0.45	(0.02)
Six	Low-culprit-probability	0.21	(0.02)	0.29	(0.02)
	Neutral	0.28	(0.03)	0.45	(0.02)

Values in parentheses are standard errors.

Next, we tested whether low-culprit-probability instructions reduce the probability of guessing-based selection (parameter *g*) compared to neutral instructions. Table 3 shows that the estimate of parameter *g* is lower in the low-culprit-probability-instruction group than in the neutral-instruction group. Imposing on the base model the additional restriction that parameter *g* did not differ as a function of whether low-culprit-probability or neutral instructions were used led to a significant decrease in model fit for both the three-person-lineup group, Δ*G*^2^(1) = 28.81, *p* < 0.001, and the six-person-lineup group, Δ*G*^2^(1) = 42.21, *p* < 0.001, implying that the equality restriction is incompatible with the data. This replicates the results of Experiment 1 and leads to the conclusion that low-culprit-probability instructions lead to a lower probability of guessing-based selection compared to neutral instructions. The restriction that parameter *dP* did not differ as a function of whether low-culprit-probability or neutral instructions were used did not lead to a statistically significant decrease in model fit compared to the fit of the base model for both the three-person-lineup group, Δ*G*^2^(1) = 1.18, *p* = 0.278, and the six-person-lineup group, Δ*G*^2^(1) = 3.72,* p* = 0.054, implying that the equality restriction is compatible with the data. This replicates the results of Experiment 1 and leads to the conclusion that culprit-presence detection does not differ as a function of the lineup instructions. It can thus be concluded that the previously found effects of low-probability instructions on the latent processes underlying eyewitnesses’ responses to lineups^20^ are robust and can be replicated.

#### Observable responses: Effects on the rates of innocent-suspect identifications and culprit identifications

**Figure 3:**
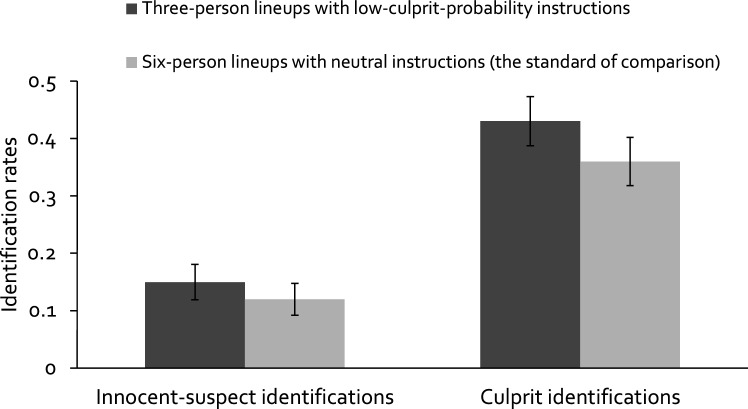
Rates of innocent-suspect identifications (left side) and rates of culprit identifications (right side) for the three-person lineups with low-culprit-probability instructions and for the six-person lineups with neutral instructions (the standard of comparison) in Experiment 2. Error bars represent the 95 % confidence intervals.

The rates of innocent-suspect identifications in three-person lineups with low-culprit-probability instructions and six-person lineups with neutral instructions are presented in Fig. 3 (left side). Three-person lineups with low-culprit-probability instructions were associated with a rate of innocent-suspect identifications close to the rate observed in six-person lineups with neutral instructions. A two-proportion *z*-test showed that there was no statistically significant difference between these groups in the rate of innocent-suspect identifications, *z* = 1.53, *p* = 0.126. This replicates the results of Experiment 1 and leads to the conclusion that the two rates are equal.

The rates of culprit identifications are also presented in Fig. 3 (right side). Three-person lineups with low-culprit-probability instructions were associated with a higher rate of culprit identifications than six-person lineups with neutral instructions. A two-proportion *z*-test showed that the difference between these groups in the rate of culprit identifications was statistically significant, *z* = 2.14, *p* = 0.032. This replicates the results of Experiment 1 and leads to the conclusion that the rate of culprit identifications is higher for three-person lineups with low-culprit-probability instructions compared to six-person lineups with neutral instructions.

### Discussion

The results of Experiment 2 largely replicate those of Experiment 1. With regard to the latent processes underlying eyewitness responses, the results confirm that the probability of culprit-presence detection is higher in smaller compared to larger lineups. In addition, low-culprit-probability instructions lead to a lower probability of guessing-based selection compared to neutral instructions.

With regard to observable responses, the results confirm the prediction that combining three-person lineups with low-culprit-probability instructions should be associated with a low rate of innocent-suspect identifications that does not differ from the rate observed in six-person lineups with neutral instructions. The results also confirm the prediction that the rate of culprit identifications should be higher in three-person lineups with low-culprit-probability instructions than in six-person lineups with neutral instructions.

## General discussion

Here we built on, and largely replicated, earlier findings showing that smaller lineups are associated with a higher probability of culprit-presence detection and a lower probability of guessing-based selection than larger lineups^19^. In the present experiments, the model-based analyses confirmed that the probability of culprit-presence detection is higher in smaller compared to larger lineups. This has to be counted as an advantage of smaller compared to larger lineups in that it seems desirable that lineup procedures support the detection of the culprit. The probability of guessing-based selection was lower in smaller compared to larger lineups in sequential (Experiment 1) but not in simultaneous (Experiment 2) lineups. Guessing-based selection leads to the identification of culprits and innocent suspects with a sampling probability of 1 ÷ *n* that is inversely related to the lineup size *n*. A useful aspect of the 2-HT eyewitness identification model (Fig. 1) is that it contains transparent assumptions about how these latent processes concur and bring about overt responses such as innocent-suspect identifications and culprit identifications. The insights gained based on the 2-HT eyewitness identification model and explicated in more detail in the introduction have laid open why a higher probability of culprit-presence detection in smaller compared to larger lineups may, among other factors, cause a higher rate of culprit identifications, and they also helped to clarify why, despite a lower probability of guessing-based selection in smaller compared to larger lineups, the inverse relationship between the sampling probability 1 ÷ *n* and the lineup size *n* caused observable rates of innocent-suspect identifications to be higher in smaller compared to larger lineups in previous experiments^19^.

However, the 2-HT eyewitness identification model is a helpful tool not only to understand why these phenomena occur but also to identify a possible solution to the problem of the increased rate of innocent-suspect identifications in smaller compared to larger lineups. As explicated in the introduction, if it would be possible to markedly reduce parameter *g* even below the level achieved by reducing the lineup size, then the rate of innocent-suspect identifications in smaller lineups should be comparatively low, ideally at least as low as the rate of innocent-suspect identifications in larger lineups with neutral instructions. One possible measure to achieve this reduction in parameter *g* is to provide lineup instructions that discourage guessing-based selection, for instance by insinuating that the culprit is unlikely to be in the lineup^3,20,30^. At the same time, the rate of culprit identifications should remain higher in smaller than in larger lineups. The results of both experiments reported here confirm these predictions. The rate of innocent-suspect identifications was the same in smaller lineups with instructions that discourage guessing-based selection and in larger lineups with neutral instructions (the standard of comparison). At the same time, the rate of culprit identifications was higher in smaller lineups with low-culprit-probability instructions than in larger lineups with neutral instructions.

At a more abstract level, the present findings demonstrate the usefulness of the 2-HT eyewitness identification model not only for measuring the latent processes underlying eyewitnesses’ responses to lineups^18,19,21^ but also for gaining insights into how these processes may be affected with the goal of potentially improving the outcomes of lineup procedures. The present results show that these insights can be used to generate testable predictions about observable responses. These predictions were confirmed in the experimental tests reported here. Going beyond these specific experimental tests, it may seem tempting to assume that the outcomes of lineup procedures can generally be improved by combining a small lineup size with instructions that discourage guessing-based selection. Here we must sound a note of caution. Whereas it may indeed turn out to be possible to improve the outcomes of lineup procedures in that way, it is at this stage far from clear how well the findings reported here can be generalized. For instance, it is not yet clear what happens if the eyewitnesses’ memory for the culprit is extremely poor or extremely good, if lineups are unfair, if lineup sizes differ from the ones investigated here (with showups clearly being out of the question^31^), if the instructions designed to discourage guessing-based selection are different from the ones used here and so on. These questions and many more will all have to be answered in future studies. The study reported here can thus only be the beginning of a larger research effort.

## Data Availability

The files with the frequency data and the equation files needed for the model-based analyses are available at https://osf.io/gcm8x/.
